# Foreign Body Induced Neuralgia: A Diagnostic Challenge

**DOI:** 10.1155/2013/352671

**Published:** 2013-05-29

**Authors:** S. Padmashree, Chaitra H. Ramprakash, Rema Jayalekshmy

**Affiliations:** Department of Oral Medicine & Radiology, Vydehi Institute of Dental Sciences and Research Centre, No. 82, EPIP Area, Whitefield, Bangalore 560 066, India

## Abstract

Neuropathic pain is caused by neural injury or painful states associated with either peripheral or central nerve injury. One of the aetiologies of this type of pain is iatrogenic trauma. This case highlights the features of peripheral neuropathic pain caused by foreign body left in the mental foramen following a previous surgical procedure. The foreign body was detected on routine radiographic evaluation. Once the foreign body was removed by surgical intervention, the pain resolved. This stresses the importance of routine radiographic evaluation in proper diagnosis and treatment planning in the management of neuropathic pain. This paper also sheds light on the role of iatrogenic mechanical cause of peripheral neuropathic pain and warrants a tough degree of caution on the part of oral clinicians.

## 1. Introduction

Neuropathic pain is caused by neural injury or painful states associated with either peripheral or central nerve injury. It is defined as pain initiated or caused by a primary lesion or dysfunction in the nervous system [1]. Neuropathic pain felt in the distribution of a nerve or nerves which is induced by the presence of a foreign body is called the foreign body neuralgia. Traumatic implantations have been published with different objects like the fish bone [2], tooth, or metallic restorations [3]. There are multiple causes of foreign body entrapment. It can occur when the soft tissues are lacerated as in the case of penetrating injuries during accidents, which can give rise to a foreign body granuloma [3]. The other causes may be in the form of an overfilling root canal restoration, fractured root canal filling instrument [4], fragment of the restoration left behind in the area during extraction, which are iatrogenic in origin. These entrapped foreign bodies can impinge the nerve and cause either paroxysmal or continuous type of pain or paresthesia, which can be felt in the area of the distribution of that nerve.

This case highlights the features of foreign body neuralgia which posed a diagnostic challenge at the clinical level and was unraveled later. 

## 2. Case Report

A 54-year-old female patient presented with a complaint of pain in the right lower back tooth region since 6–9 months. Pain was continuous, dull aching type localized to the right lower jaw region. It increased on chewing food, talking and decreased slightly on taking medication. She gave a history of pain 4 years ago which was sharp, intermittent, and localized in the same region. It increased on chewing food and reduced on massaging the area on the right side of the lower jaw and gradually with time. Hence, she underwent extraction of all the posterior teeth on the right side of the jaw which gave her no relief from the pain. Visiting another dentist, with the same complaints, she was diagnosed with trigeminal neuralgia and medications were prescribed. Even after one year of medication, she did not find relief from the pain. That is when visiting another dentist was decided around 3 years ago. The mental nerve block resulted in pain reduction; hence, she was advised to undergo mental nerve avulsion, which she underwent under local anesthesia. 

**Figure 1:**
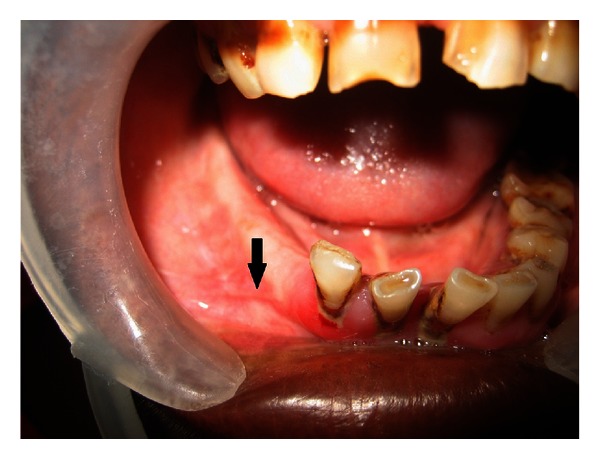
Intraoral photograph showing the previous surgical site which was firm and tender on palpation.

After the surgery, there was complete reduction in pain, but after 2 months of the procedure, she developed dull aching, burning type of pain in the same region. Hence, she visited our institution with the complaint of pain in the right mandibular premolar region. The patient had no contributing medical or dental history. On general physical examination, no abnormalities were detected and all the vital signs were within normal limits. The TMJ evaluation revealed no abnormalities. On extraoral palpation, paresthesia was detected in the lower lip on the right side and a part of the chin and the lower part of the cheek on the right side. On intraoral examination, a fibrosed scar was seen in the region of the right mandibular premolar on the alveolar mucosa about 2-3 mm above the buccal vestibule, measuring about 0.5 cm in diameter. The scar was firm and tender on palpation; it could be pinched off from the underlying bone (Figure 1). The general periodontal status of the patient was poor along with generalized enamel hypoplasia secondary to enamel fluorosis.

**Figure 2:**
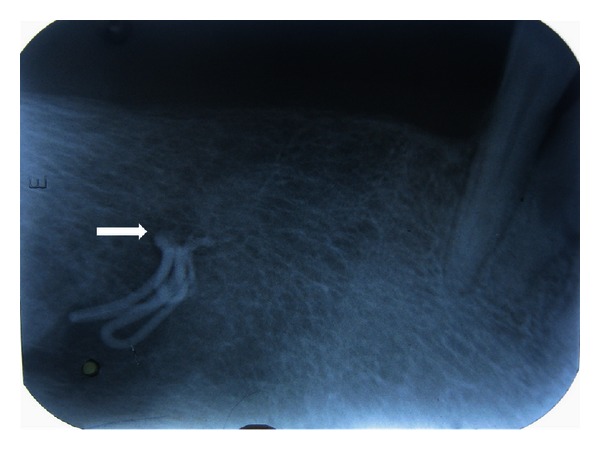
Intraoral radiograph showing the radiopaque foreign body close to the mental foramen.

**Figure 3:**
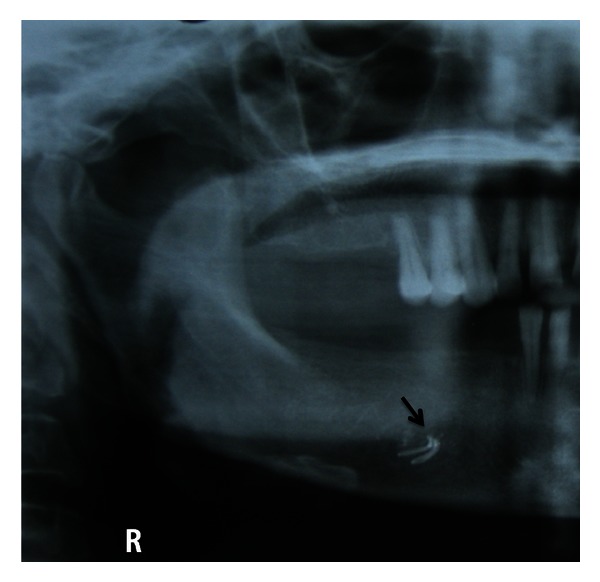
Part of the OPG showing the presence of radiopaque foreign body in the region of right mental foramen.

Taking into consideration the complaint and the presenting symptoms, arriving at a diagnosis was a challenge, as the features were atypical of trigeminal neuralgic pain and no lesion was evident except for the scar which was caused due to the previous surgical intervention. Therefore, provisionally, it was diagnosed as neuropathic pain on the right mental region of the jaw. Traumatic neuroma and infected and retained root stumps in the right premolar region were considered in differential diagnosis, taking into consideration the previous surgical intervention in terms of mental nerve avulsion and extraction of the teeth. As in both cases, the patient may present with continuous type of pain localized to the area of origin. To assess the condition of the mandibular canal and the premolar region of the jaw radiologic examination was essential. Interestingly, on intraoral periapical radiograph, a radiopaque material was detected in the right premolar region well within the substance of the mandible, in the region of the mental foramen on the right side (Figure 2). The same was confirmed on the OPG which revealed the foreign material and the outline of the mental foramen which was not clearly made out on the right side (Figure 3). The importance of radiologic evaluation is quite evident in helping us to arrive at a final diagnosis of foreign body neuralgia in the right mental foramen.

**Figure 4:**
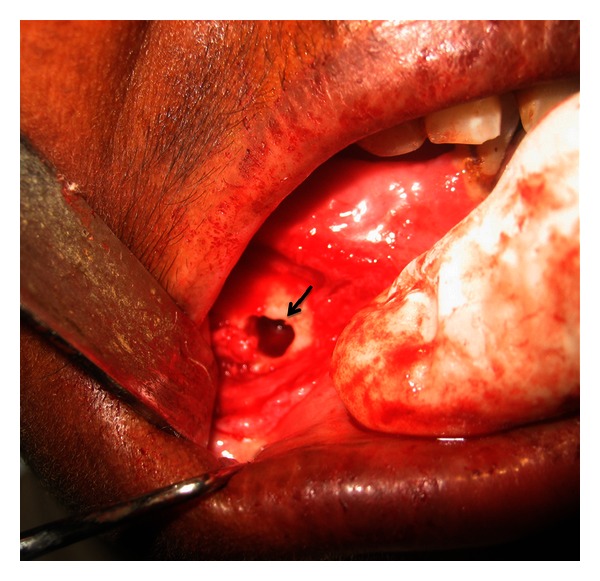
Surgical exploration of the right mental foramen done under local anesthesia.

**Figure 5:**
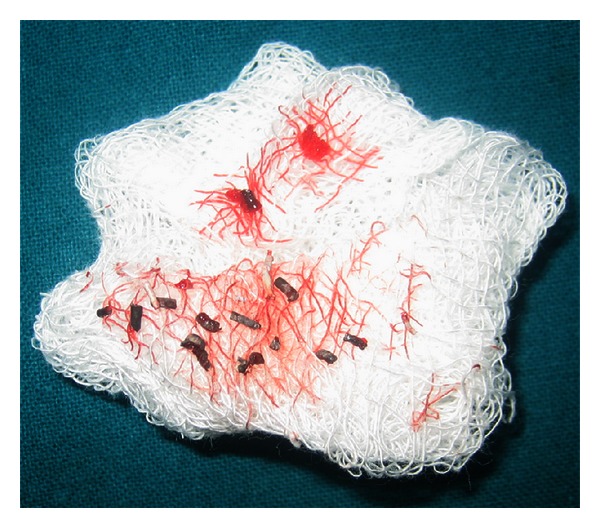
The photograph of the retrieved gutta-percha and silver points.

**Figure 6:**
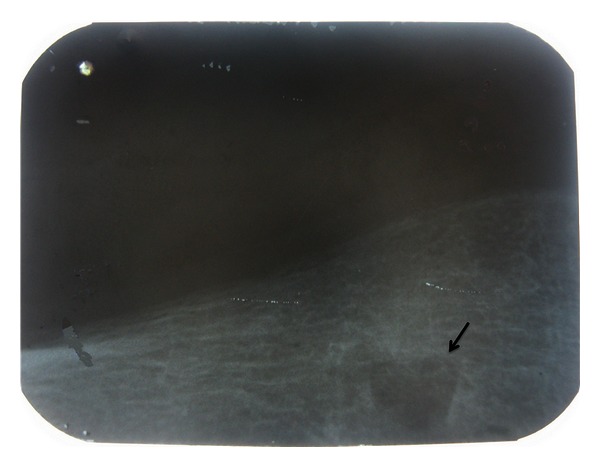
Postsurgical intraoral periapical radiograph showing the enlarged mental foramen with complete absence of the foreign material.

**Figure 7:**
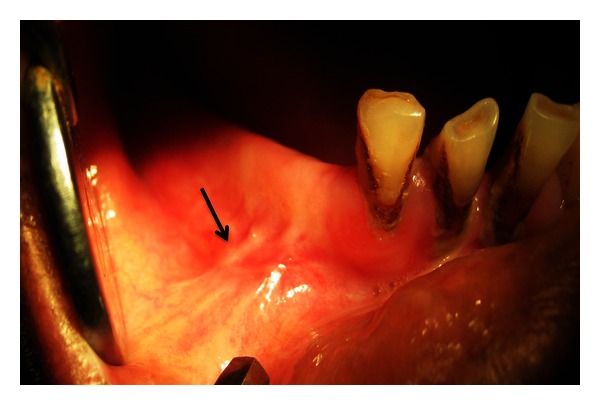
The follow-up intraoral photograph showing complete healing of the surgical site.

Surgical intervention was planned to explore the right mental foramen under local anesthesia (Figure 4) and the foreign material was retrieved, which consisted of silver points, gutta-percha points and restorative material (Figure 5). Postsurgical radiograph reveals enlarged mental foramen and complete absence of the radiopaque material (Figure 6). The surgical site was sutured and the patient was advised to take an analgesic for the day if required and to stop all the other medication. During her first week of the postsurgical recall for suture removal, healing of the surgical site was satisfactory. She was reviewed after three months which revealed completely healed surgical site (Figure 7) and more importantly the patient was free of pain and continues to be on periodic follow up. 

## 3. Discussion

Neuropathic pain as defined by International Association for the Study of Pain is a type of pain initiated or caused by a primary lesion or dysfunction in the nervous system [1]. It is caused by neural injury or painful states associated with either peripheral or central nerve injury. Types of neuropathic pain can be classified based on anatomic location, etiology, and the type of pain experienced by the patients (Table 1).

 It has multifactorial etiology, which includes infections, trauma, metabolic abnormalities, chemotherapy, surgery, radiotherapy, neurotoxins, nerve compression, inflammation, and tumor infiltration. The continuous type of neuropathic pain can be caused by foreign bodies' entrapments in the mandibular and submandibular regions which are quite common. These intraosseous entrapments can lead to inflammation and foreign body granuloma formation [3]. This can occur following entrapment of any of the following materials, which could be iatrogenic in origin during the course of any treatment procedures [5]: like amalgam filling, broken injection needle, air turbine bur, endodontic filling material, broken toothbrush bristle, or sometimes tooth displacement.

Foreign body (inflammatory) reaction or injury of inferior alveolar nerve (IAN)/mental nerve may be classified into metallic or nonmetallic, temporary or permanent, and chemicomechanical or thermal in nature [6]. This entrapped foreign body can give rise to paresthesia. Paresthesia is defined as a burning or prickling sensation or partial numbness caused by neural injury [7, 8]. Patients have described it as warm, cold, burning, aching, prickling, tingling, pins and needles, numbness, itching, and formicating. Paresthesia in dentistry can be caused by local or systemic factors. Local factors include traumatic injuries such as mandibular fractures, expanding compressive lesions (benign or malignant neoplasia and cysts [9]), impacted teeth, local infections (osteomyelitis, periapical [10], and peri-implant infections), iatrogenic lesions after teeth extractions, anesthetic injection, endodontic therapy [11] (overfilling and apical surgery), implantology, orthodontic surgery, and preprosthetic surgery. According to Seddon's classification for peripheral nerve injury [12], there are three basic injuries: neurapraxia, axonotmesis, and neurotmesis. Neurapraxia is a temporary conduction block after mild compression of the nerve trunk (i.e. paraesthesia or dysaesthesia of the lip and chin region in case of IAN/mental nerve). Axonotmesis, a more serious condition, results from degeneration of the afferent fibers as a result of internal/external irritation (i.e. anaesthesia), while in neurotmesis the nerve is completely severed which results in permanent paraesthesia. The main mechanism of injury of peripheral nerves is compression. Compression of the arterial blood supply to the nerve results in increased vascular permeability, edema, and ischemia, and the amount of oxygen delivered to the nerve is thereby reduced [13]. The classic response in cases of neuropraxia is paresthesia, but axonotmesis may also occur if the compression lasts long enough; in these cases the recovery can take up to 1 year.

Our case posed a diagnostic challenge as the patient's clinical presentation was atypical of trigeminal neuralgia. If the fibrosed scar was ignored and the high doses of medication were prescribed to manage the neuropathic pain, it would have done more harm to the patient than to help her. The simple routine intraoral radiological examination revealed the actual cause of the pain, thereby aiding us in our diagnosis of a foreign body induced neuralgia. The removal of the cause is the primary management. Hence, we were able to help the patient get rid of her pain with the help of imaging. This case emphasizes the need for routine radiological investigations in the diagnosis of the cases of peripheral neuralgia. 

## 4. Conclusion

Patients presenting with sensory symptoms in the distribution of the IAN/mental nerve area should be assessed carefully by proper history recording, taking into consideration any previous surgical intervention. This should be followed by detailed examination and radiological investigation. Diagnosticians should be aware of the presence of a foreign body which can give rise to neuropathic pain. Every effort should be made to avoid any iatrogenic entrapments like the overfilling of root canals, apical surgery, traumatic extraction, fractured fragments of the restoration, and so forth. This case further emphasizes the importance of the radiographic evaluation in diagnosing foreign body induced neuralgia.

## Figures and Tables

**Table 1:** Classification of the types of neuropathic pain [1].

Based on anatomic location and etiology	Based on type of pain
(i) Peripherally mediated (ii) Centrally mediated (iii) Metabolic polyneuropathies	(i) Paroxysmal/episodic (a) Neurovascular pains (b) Neuralgic pains (ii) Continuous
